# The Tubercle-Bacillus in Hens

**Published:** 1883-10

**Authors:** 


The Tubercle-Bacillus in Hens.—Dr. Ribbert, of Bonn, finds that tuberculosis sometimes attacks hens, and that the disease may even become epidemic in a flock.

He states in the *Deutsche Medicine, Wochenschrift* that the bacilli of tuberculosis are invariably to be found chiefly in the intestinal wall, spleen and liver. He further asserts as the result of his experiments that the bacilli are distributed chiefly through the virus and are to be seen in large numbers about venous walls.

A flock of hens suffering from tubercular disease can not be a very safe addition to a farm-yard.

---

The attention of our readers is called to the article on Comparative Menstruation by Alfred Wiltshire, M.D. It is only very recently that human physicians and scientists have turned their attention and researches to the development and study of comparative science.

The article which we have reprinted from the *Veterinary*

*Journal* is carefully and thoroughly worked up and illustrates the kind and character of the work that is being done by those interested in the laws of life and development as manifested in the lower animals.

Dr. Wiltshire is recognized as an authority, and his views upon so interesting a subject as the Physiology of Menstruation should command attention notwithstanding that they may seem to some to conflict with the traditional orders on this subject. Contributions of a similar character on any subject by those competent to write them will always find space in our original department.

